# Improving skin picking diagnosis among Brazilians: validation of the Skin Picking Impact Scale and development of a photographic instrument^☆^^☆☆^

**DOI:** 10.1016/j.abd.2018.10.001

**Published:** 2019-09-30

**Authors:** Alice Castro Menezes Xavier, Camila Maria Barbieri de Souza, Luís Henrique Fernandes Flores, Clarissa Prati, Cecilia Cassal, Carolina Blaya Dreher

**Affiliations:** a Postgraduate program of Health Sciences, Federal University of Health Sciences of Porto Alegre, , Porto Alegre, RS, Brazil; b Medical School, Federal University of Health Sciences of Porto Alegre, Porto Alegre, RS, Brazil; c Outpatient Dermatology Clinic, Pontifical Catholic University of Rio Grande do Sul, Porto Alegre, RS, Brazil; d Outpatient Clinic of Psychodermatology, Outpatient Clinic of Hygienic Dermatology, Rio Grande do Sul State Health Department,, Porto Alegre, RS, Brazil; e Department of Clinical Medicine – Psychiatry, Medical School, Federal University of Health Sciences of Porto Alegre, , Porto Alegre, RS, Brazil; f Department of Psychiatry and Forensic Medicine, Medical School, Federal University of Rio Grande do Sul, Porto Alegre, RS, Brazil

**Keywords:** Photography, Psychometrics, Symptom assessment, Validation studies

## Abstract

**Background:**

Skin picking disorder is a prevalent disorder frequently comorbid with depression and anxiety, which is underdiagnosed mainly by dermatologists. Assessment of skin picking disorder is based on instruments influenced by the awareness about skin picking disorder and comorbid symptoms. To date, there is no validated instrument for Brazilian individuals nor an instrument to evaluate the severity of skin lesions in an objective way.

**Objectives:**

Validate the Skin Picking Impact Scale for Brazilian Portuguese and create a photographic measurement to assess skin lesions.

**Methods:**

The sample was assessed through the Skin Picking Impact Scale translated into Brazilian Portuguese, the Hamilton Anxiety Scale, the Beck Depression Inventory, and the Clinical Global Impression Scale. The patients’ skin lesions were photographed. Photos were evaluated regarding active excoriation, crust/bleeding, exulceration, and linear lesions.

**Results:**

There were 63 patients included. The Skin Picking Impact Scale translated into Brazilian Portuguese had good internal consistency (Cronbach's alpha = 0.88), which tests of goodness-of-fit, showing a suitable model. The reliability of photographic measurement was 0.66, with a high internal consistency (Cronbach's alpha = 0.87). Photographic measurement was not correlated with the Skin Picking Impact Scale, the Clinical Global Impression Scale, or comorbid symptoms.

**Study limitations:**

Lack of a previously validated instrument to evaluate dermatillomania in the Brazilian population for comparison.

**Conclusion:**

The Skin Picking Impact Scale validated in Brazilian Portuguese is a good instrument to evaluate skin picking disorder. Photographic measurement is a consistent way of assessing skin lesions, but it does not reflect the impact of skin picking disorder on the individual's life.

## Introduction

Skin picking disorder (SPD), also known as excoriation disorder or dermatillomania, affects up to 5.4% of general population and up to 30% of psychiatric patients.^1^ It was included as a formal diagnosis in the Diagnostic and Statistical Manual of Mental Disorders, Fifth Edition (DSM 5), characterized by the following: recurrent picking at the skin resulting in skin lesions; attempts to stop picking; clinically significant distress, and absence of another cause for the habit.^2^ SPD severity is related to impairment in quality of life, and is correlated with anxiety disorder (up to 48%), major depression (8–28%), and substance abuse (14–36%).^3^

Although it is a psychiatric disorder, the majority of patients first seek help with a dermatologist, and differential diagnosis should be made with delusional parasitosis, prurigo simplex, subacute Hebra and scabies.^4^ Among patients with SPD who seek help, only 53% receive the correct diagnosis, made by dermatologists in 2.5% of cases and by psychiatrists in 56%.^1^ SPD is a chronic disorder and the habit usually is automatic, with only 24% of individuals reporting full awareness prior to picking.^5^ Besides the nails, patients can use pins or tweezers to manipulate their skin, and 80% have notable tissue damage.^1^

The severity of SPD and the response to treatment have been evaluated with different instruments: clinician-reported measures, self-reported measures of SPD severity, and self-reported measures of SPD impact. The Yale-Brown Obsessive Compulsive Scale Modified for Neurotic Excoriation (NE-YBOCS) is a reliable clinician-reported instrument that evaluates thoughts and behaviors related to skin picking during the last seven days, but does not specifically assess the social distress caused by the disease.^6^ Among the self-reported measures, the skin picking scale is a moderately reliable instrument that assesses SPD severity during the past week and that has been correlated with depressive symptoms.^7^, ^8^ The Skin Picking Symptom Assessment Scale is a less-used, reliable, self-reported instrument to evaluate SPD severity during the last seven days.^6^ The Skin Picking Reward Scale is a reliable instrument to assess the aspects of “liking” and “wanting” the SPD habit, without definition of the time of symptoms evaluation, substantially influenced by the self-reflection regarding the habit.^6^ All these measures evaluate the frequency of this habit but not the distress caused. The Skin Picking Impact Scale (SPIS) is the only scale that evaluates the psychological impact of this disorder; it is a patient-rated scale with high reliability, evaluating symptoms during the last seven days, with higher scores considered more severe impact. The SPIS has been correlated with depressive and anxious symptoms.^9^

The available instruments to assess SPD depend of the patient's awareness about the habit, which is not common. The NE-YBOCS also depends on clinician knowledge about emotional distress, which is not easy for dermatologists, who are generally the first professional accessed by patients suffering from SPD. Self-applied instruments are also influenced by emotional aspects of the patient: anxiety, depression, and social avoidance, which is frequent in SPD. In addition, the body area affected directly influence the scales, with patients who pick their faces generally having a high score because of higher self-esteem impact.^10^ The majority of instruments evaluate the habit during last seven days, although it is known that SPD is a chronic disease and that the time to heal severe wounds would be longer. Even though there are different methods of evaluation for SPD, no study has evaluated skin-picking severity through different approaches at the same time.

There is a lack of an objective way to assess SPD symptoms not influenced by patients’ awareness of the habit or other emotional conditions. A potential advantage of a photographic evaluation is the reduction of reporting bias and recognition of the severity of the skin lesions. To the authors’ knowledge, no photographic measure has been validated to assess SPD severity, with only two studies having used photographs to evaluate SPD, without validity or reliability of the measure being published.^11^, ^12^

Despite the high prevalence of SPD, to the authors’ knowledge no instruments to assess this pathology have been validated to be used in the Brazilian population. As the impact of SPD on a patient's life is important information about the individual's suffering, and because the SPIS, a highly reliable scale, was previously translated to Brazilian Portuguese (Ferrão Y.A. and Bedin, N.R., data not published), the validation of this instrument for Brazilian population could be a valuable way to assess SPD.

The aim of this study is to validate the SPIS for the Brazilian population and to create a photographic instrument (PI) to assess SPD severity, applicable by dermatologists. This study works with the hypothesis that the SPIS and the PI can be reliable and easily used in clinical practice, with PI reflecting more directly the severity of SPD, independently of comorbid symptoms. In this way, the diagnostic rates of SPD can be increased.

## Methods

### Study design and participants

This was a cross-sectional study in which participants were enrolled through public advertising in the period between July 2014 and January 2018. The inclusion criterion was a diagnosis of SPD according to a diagnostic interview structured by the DSM 5.^2^ The exclusion criteria were current psychotic disorder, moderate or severe intellectual disability (two individuals excluded), or suicide risk (one individual excluded) as assessed by clinical interview conducted by trained psychiatry residents. Individuals who met exclusion criteria received referrals to appropriate community resources.

The primary endpoint of the study was to validate the SPIS for the Brazilian population and to create a valid and reliable objective PI to assess SPD severity. Secondary outcomes were the correlation between PI and the instruments that assess the comorbidities and impact on life of SPD, such as the Beck Depression Inventory (BDI), the Hamilton Anxiety Scale (HAM-A), and the Clinical Global Impression Scale (CGI).^13^, ^14^, ^15^, ^16^, ^17^

### Procedure and measures

Psychiatric diagnostic interviews were conducted by a psychiatry resident and discussed with a senior researcher. A check-list of SPD symptoms according to the DSM 5 was applied^2^ and the Mini International Neuropsychiatric Interview (MINI) translated to Brazilian Portuguese was used to assess psychiatric comorbidities.^2^, ^18^, ^19^ After the inclusion in the study, the baseline assessment was conducted by two trained evaluators with a high inter-rater reliability (kappa coefficient = 0.9), applying the BDI, the HAM-A, the CGI, and the SPIS translated to Portuguese.^9^, ^14^, ^15^, ^17^ The evaluators also took photos of patient's regions with active lesions, using an iPhone 7 camera with 12 megapixels, without zoom, at a distance from camera to skin of 7 to 11 inches, at a 90° angle.

The Portuguese version of the 21-item BDI, validated for Brazilian subjects, was used to assess depressive symptoms during the last week, with score varying from 0 to 63, and with higher scores representing more severe symptoms.^13^, ^14^ Anxiety symptoms were evaluated through the HAM-A, a questionnaire applied by the interviewer validated to Portuguese, with 13 self-reporting domains and one domain of interviewer impression, with varying scores from 0 to 56, and with higher scores representing more severe symptoms.^15^, ^20^ The psychiatric evaluation of SPD impact was performed with the CGI, applied by the interviewer to assess the impact of the disease on the patient's life, with scores varying from 1 (asymptomatic) to 7 (very severe impact).^16^ The impact of SPD was evaluated with the Skin Picking Impact Scale (SPIS), a self-applied questionnaire translated to Portuguese that evaluates symptoms of the last week using ten items, with score varying between 0 and 50, with a higher score representing more severe impact.^9^

The PI was developed by two dermatologists with experience treating SPD. In order to evaluate lesions that reflect the active habit of skin picking instead of scars from old injuries, the instrument evaluates lesions severity in four categories: excoriation; crust/bleeding; exulceration, and linear lesions. Each category was scored on a Likert scale of increasing severity from 0 to 10, taking into account the severity of each lesion and the number and size of the lesions. All the photos were analyzed by the two blinded dermatologists, who applied the PI to each image. Images of the same patient were scored separately and then an arithmetic mean of all images was created by each evaluator. Another arithmetic mean was created using the final score that the specific dermatologist gave to each patient, resulting in one average score for each individual.

### Statistical analysis and ethical considerations

Statistical analysis was performed using the software R 3.3.1. The data were analyzed for normal distribution using the Kolmogorov-Smirnov test. The chi-squared test was used to compare categorical variables. Linear correlations were made using Spearman's correlation (*r*_s_). The exploratory factor analysis was performed with oblique rotation. The internal consistency of the scale was examined using Cronbach's alpha coefficient. The instrument's structure was tested using confirmatory factor analysis, which offers a variety of tests and indices to assess the goodness-of-fit of the data. The indices used included chi-squared goodness-of-fit statistics, the root-mean-square error of approximation (RMSEA), and the Tucker-Lewis Index (TLI), and as recommended by current literature, a good model was considered to have an RMSE < 0.06, a chi-squared goodness-of-fit test with *p* > 0.05, and a TLI > 0.95.^21^ The reliability of the PI was calculated using the inter-observer concordance, using the intraclass correlation coefficient in the two-way random effect model, with a single rater and absolute agreement, and with a value equal to or greater than 0.4 considered a moderate concordance, and a value equal to or greater than 0.8 considered a perfect concordance.^22^, ^23^ The level of significance was set at *p* < 0.05. Data were controlled for comorbid depressive and anxious symptoms.

All participants signed an informed consent to participate in the research. This study is in accordance with the Guidelines and Norms Regulating Research Involving Human Beings (Resolution No. 466/12), following the ethical principles of the Declaration of Helsinki. The study was approved by the Ethics and Research Commission (CEP) under protocol No. 1,197,672.^24^

## Results

The study included 63 patients. Table 1 depicts the epidemiological and sociodemographic characteristics of the sample.

**Table 1:** Sociodemographic and clinical characteristics of the sample

	Total sample (*n* = 63)
*Age (years), mean (standard deviation)*	36.30 (13.72)
*Female gender, n (%)*	56 (88.9)
*Schooling (years), mean (standard deviation)*	14.43 (3.53)
*Caucasian, n (%)*	54 (85.7)

*Marital status, n (%)*	
Single	33 (52.4)
Married	19 (30.2)

*Religion, n (%)*	
Catholic	21 (33.3)
Agnostic	14 (22.2)

*Occupation, n (%)*	
Working	25 (39.7)
Student	19 (30.2)

*Income (US$), median (minimum–maximum)*	598.8 (0‒8.383.2)
*Trigger situation, n (%)*	21 (33.3)
*Age of SPD onset, median (minimum*–*maximum)*	14.5 (4‒64)

*Previous treatment, n (%)*	
None	38 (60.3)
Medication	11 (17.5)

*Family history of SPD, n (%)*	16 (25.4)
*Family history of psychiatric disease, n (%)*	26 (41.3)

*Comorbidities, n (%)*	
Current depressive episode	16 (25.4)
Past depressive episode	13 (20.6)
Dysthymia	4 (6.3)
Bipolar affective disorder	6 (9.5)
Panic disorder	18 (28.6)
Agoraphobia	10 (15.9)
Social anxiety	6 (9.5)
Obsessive compulsive disorder	2 (3.2)
Post-traumatic stress disorder	3 (4.8)
Alcohol dependence	1 (2)
Nervous bulimia	2 (3.2)
Generalized anxiety disorder	25 (39.7)

*Psychotropic treatment, n (%)*	
Selective serotonin reuptake inhibitor	25 (39.68)
Dual antidepressant	8 (12.69)
Tricyclic antidepressant	2 (3.17)
Atypical antidepressant (trazodone and bupropion)	2 (3.17)
Mood stabilizer	9 (14.28)
Benzodiazepines/hypnotics	5 (7.93)
Antipsychotics	4 (6.34)
Methylphenidate	1 (1.58)

*Clinical scales*	
SPIS, median (minimum–maximum)	27.5 (3–48)
CGI, median (minimum–maximum)	5 (2–7)
BDI, mean (standard deviation)	15.66 (10.65)
HAM-A, mean (standard deviation)	28.41 (14.68)

SPD, skin picking disorder; SPIS, Skin Picking Impact Scale; CGI, Clinical Global Impression; BDI, Beck Depression Inventory; HAM-A, Hamilton Anxiety Scale.

**Figure 1:**
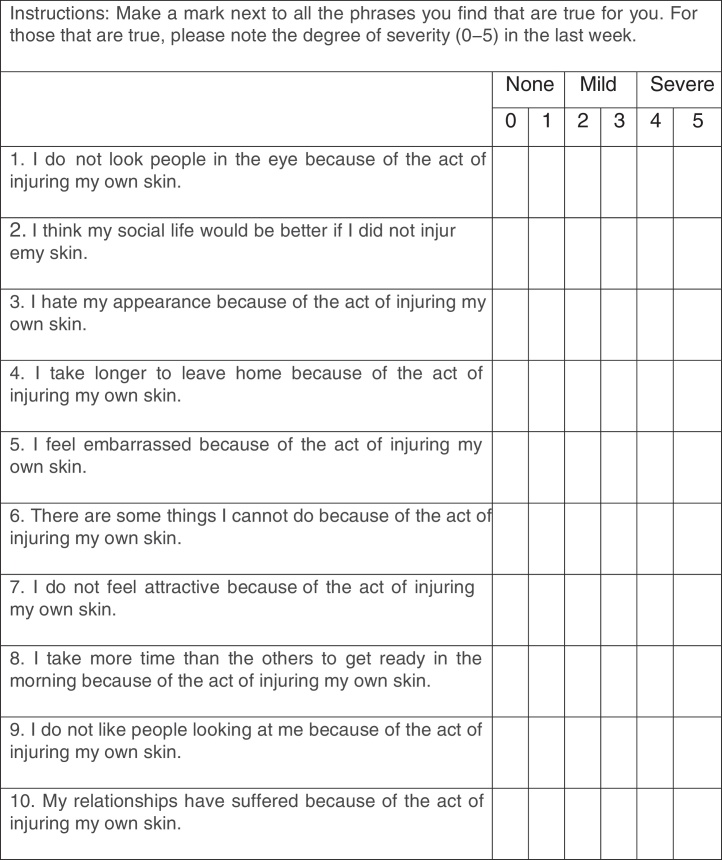
Skin Picking Impact Scale validated for Brazilian Portuguese.

The validation of the SPIS resulted in a Cronbach's alpha of 0.88. The exploratory factor analysis showed a two-factor scale, resulting in a RMSEA < 0.00001, with chi-squared goodness-of-fit test = 21.61; *p* < 0.71, and TLI = 1.034. Fig. 1 shows the translated SPIS.

**Figure 2:**
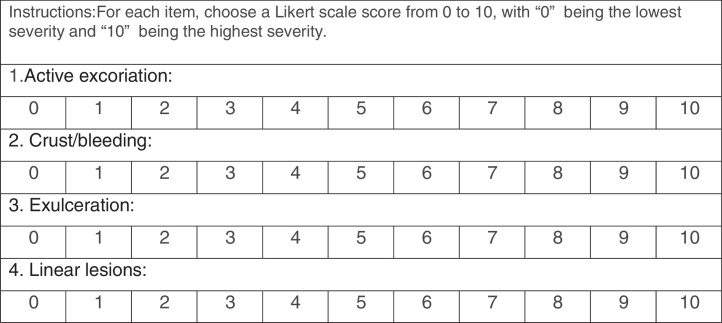
Photographic instrument to assess skin lesions caused by skin picking disorder.

The PI created by the two experts is shown in Fig. 2. Cronbach's alpha was 0.87 and the reliability was moderate, with intra-class correlation of 0.6. Table 2 shows the detailed information about the PI scores and reliability values. The exploratory factor analysis found a one-factor model, with the sub-item ‘Active Excoriation’ having the largest factorial load, of 0.978.

**Table 2:** Mean photographic instrument scores of the sample and their intra-class correlation between evaluators

Item	Evaluator 1	Evaluator 2	ICC	ICC (95%)
Active excoriation	2.4	2.83	0.718	[0.559; 0.825]
Crust/bleeding	1.06	1.76	0.489	[0.196; 0.687]
Exulceration	0.72	1.6	0.438	[0.091; 0.666]
Linear lesions	0.37	0.53	0.522	[0.305; 0.688]
Total score	1.15	1.69	0.661	[0.319; 0.823]

ICC, intra-class correlation; ICC (95%), intra-class correlation and its confidence interval.

Analyzing the correlation between the instruments, the SPIS was correlated with the anxious and depressive symptoms evaluated by the HAM-A and BDI scales. However, the PI was not correlated with the CGI, nor with comorbid symptoms (Table 3).

**Table 3:** Spearman's correlation between instruments

Scale	CGI	SPIS	HAM-A	BDI	PI
CGI	1	0.69^a^	0.5^a^	0.66^a^	0.1
SPIS		1	0.53^a^	0.61^a^	0.11
HAM-A			1	0.78^a^	0.02
BDI				1	0.11

CGI, Clinical Global Impression; SPIS, Skin Picking Impact Scale; HAM-A, Hamilton Anxiety Scale; BDI, Beck Depression Inventory; PI, photographic instrument.

a *p*-Value < 0.05.

Evaluating patients according to the region of lesions, those with lesions in the face tend to have a higher CGI score than patients without, but this did not reach statistical significance (mean = 4.95, SD = 1.22 *vs.* mean = 4.32, SD = 1.29; *p* = 0.08).

## Discussion

This study validated the SPIS to be used in the Brazilian Portuguese version as a reliable instrument. The SPIS is a self-applied instrument that does not depend on clinician knowledge about SPD. Although SPD is a prevalent condition, many clinicians are not familiarized with this diagnosis and in many cases non-psychiatric professionals are the first sought by patients, leading to underestimation in the diagnosis and impact on life of SPD. Having a self-applied scale able to be used by all health professionals is a reasonable way to increase the assessment of SPD diagnosis and severity, overcoming the barrier of low referral to treatment.^1^, ^4^

In order to assess skin-picking lesions in an objective way, two dermatologists created the PI, measuring SPD based on severity of the active lesions in the skin. To date, despite having many scales to assess SPD, there is no instrument to directly evaluate the lesions caused by the habit. It is known that patients can cause severe lesions, sometimes using objects to manipulate the skin, leading to infections and in more extreme cases septicemia and death.^25^ The lesion severity is not always associated with the impact on life of SPD, as patients with small lesions in the face can have higher scores on the SPIS than patients with extensive covered lesions.^10^ The PI created in this study can be an appropriate instrument to assess lesions from SPD, improving the diagnosis of severe lesions not assessed by existing scales and facilitating earlier treatment. This instrument was easily applied by different evaluators.

As expected, the Brazilian Portuguese version of the SPIS had a positive correlation with the patients’ global clinical status and their depressive and anxious symptoms. The high rates of patients with depressive and/or anxious syndromes in this study reflect the high rates of comorbidity of SPD and depression/anxiety. The PI created had no correlation with comorbid symptoms, the global clinical status, nor the SPIS, suggesting that the severity of lesion is not associated with individual life impact. Indeed, dermatologists of this study tended to underestimate the severity of SPD impact when evaluating only skin lesions. Although not statistically significant, this study found a more severe global clinical condition in those patients with more exposed lesions, independent of the lesion severity, which is in accordance with literature.^10^

The present results should be interpreted in light of some limitations. The sample was collected through advertising, which may have generated a selection bias, as patients with low SPD life impact, despite having severe lesions, might not have felt motivated to access this service. On the other hand, patients with extremely severe SPD life impact, with associated social impairment, may not have felt in condition to spontaneously look for health assistance. The strengths of this study are the sample size, powerful in comparison with previous studies, the well-established statistical methods used, and the innovative findings.^22^, ^26^, ^27^

## Conclusion

The Brazilian Portuguese version of the SPIS is a valid and reliable instrument to assess SPD severity and its impact on life in Brazilian individuals, which is easily applied by psychiatrists and non-psychiatric clinicians. A PI to evaluate the severity of lesions caused by SPD can be applied by dermatologists and objectively reflects the lesion severity, although not reflecting the suffering of the individual. To improve SPD diagnosis, both instruments should be applied in the evaluation of the patient. Further studies are expected to assess the validity of the Brazilian Portuguese version of the SPIS when used to assess response to treatment. Also, future studies should evaluate if the PI can be applied by non-dermatologists, and if it can be a reliable way to assess the patient's improvement after treatment.

## Financial support

A grant was provided to the first author by The Ministry of Education (MEC) – *Fundação Coordenação Aperfeiçoamento de Pessoal de Nível Superior* (CAPES).

## Author's contribution

Alice Castro Menezes Xavier: Statistical analysis; approval of the final version of the manuscript; conception and planning of the study; elaboration and writing of the manuscript; obtaining, analyzing and interpreting the data; effective participation in research orientation; intellectual participation in propaedeutic and/or therapeutic conduct of the cases studied; critical review of the literature; critical review of the manuscript

Camila Maria Barbieri de Souza: Approval of the final version of the manuscript; conception and planning of the study; obtaining, analyzing and interpreting the data; critical review of the manuscript.

Luís Henrique Fernandes Flores: Approval of the final version of the manuscript; conception and planning of the study; obtaining, analyzing and interpreting the data; critical review of the manuscript.

Clarissa Prati: Conception and planning of the study; obtaining, analyzing and interpreting the data; critical review of the manuscript.

Cecilia Cassal: Conception and planning of the study; obtaining, analyzing and interpreting the data; intellectual participation in propaedeutic and/or therapeutic conduct of the cases studied.

Carolina Blaya Dreher: Statistical analysis; approval of the final version of the manuscript; conception and planning of the study; elaboration and writing of the manuscript; effective participation in research orientation; intellectual participation in propaedeutic and/or therapeutic conduct of the cases studied; critical review of the manuscript.

## Conflicts of interest

The authors declare no conflicts of interest.
